# Editorial: Emerging trends in cancer research: diagnostic and therapeutic breakthroughs

**DOI:** 10.3389/fmolb.2025.1750771

**Published:** 2025-12-02

**Authors:** Valentyn Oksenych, Oleksandr Kamyshnyi, Rostyslav Bilyy

**Affiliations:** 1 Faculty of Medicine, University of Bergen, Bergen, Norway; 2 Oslo Bioconsulting, Oslo, Norway; 3 Department of Microbiology, Virology, and Immunology, I. Horbachevsky Ternopil National Medical University, Ternopil, Ukraine; 4 Institute of Cellular Biology and Pathology “Nicolae Simionescu”, Bucharest, Romania; 5 Department of Histology, Cytology and Embryology, Danylo Halytsky Lviv National Medical University, Lviv, Ukraine; 6 Lectinotest R&D, Bucharest, Romania

**Keywords:** cancer, biomarkers, molecular diagnostics, targeted therapies, immunotherapy, personalized medicine

The Research Topic “*Emerging Trends in Cancer Research: Diagnostic and Therapeutic Breakthroughs*” synthesizes recent advances in oncology, highlighting strategies for early detection, improved prognostic tools, analysis of the complex tumor microenvironment (TME), and development of precision therapies. Despite extensive efforts, cancer remains a major global health challenge, encompassing diverse pathologies with different cellular origins. Hepatocellular carcinoma (HCC), for example, ranks as the third leading cause of cancer-related mortality worldwide, causing over 800,000 deaths annually (Alemayehu et al. and Sung et al., 2021), while cutaneous melanoma (CM) is a highly aggressive skin malignancy, with incidence rates rising dramatically in recent decades (Dudin et al. and Siegel et al., 2022). Addressing these persistent challenges requires breakthroughs in molecular science and integrated clinical strategies. The papers compiled here reflect this imperative, emphasizing minimally invasive molecular diagnostics, immunotherapy targeting the tumor microenvironment (Wan et al., 2025), and advanced genomic approaches to guide personalized treatment. Collectively, they aim to overcome key limitations in late-stage diagnosis and therapy resistance, advancing more effective and precise interventions for cancer management (Alemayehu et al.).

Significant efforts are focused on improving diagnostic and prognostic precision through molecular profiling and liquid biopsy technologies. The value of circulating biomarkers is well illustrated, exemplified by a systematic review and meta-analysis demonstrating that circulating microRNAs hold promise as non-invasive diagnostic biomarkers for hepatocellular carcinoma (Alemayehu et al.). For colorectal cancer (CRC), where early-stage symptoms are often lacking (Bray et al., 2024), novel markers like cystatin S (CST4) are emerging. CST4 demonstrates stability post-chemotherapy and significantly enhances diagnostic sensitivity for malignant colorectal lesions when combined with conventional tumor markers (CEA, CA125, CA724), representing a 28.4% increase in sensitivity over CST4 alone (Han et al.).

The increasing need for detailed genetic information drives the application of advanced sequencing methods, including Next-Generation Sequencing (NGS) and Third-Generation Sequencing (TGS), to unravel circulating tumor DNA (ctDNA) mutations in liquid biopsies (Da Silva et al.). The requirement for molecular precision is underscored by cases illustrating diagnostic pitfalls, where malignancies like pulmonary Ewing sarcoma (ES) can be misdiagnosed as more common entities such as Small Cell Lung Cancer (SCLC) based solely on histopathology, necessitating early molecular testing for accurate diagnosis and management selection (Waary et al.). Moreover, prognostic models are being refined using routinely available markers, such as assessing uric acid (UR) and the Neutrophil/Lymphocyte Ratio (NLR) in CRC. Elevated UR and NLR levels were found to be independent risk factors for bone metastasis, reflecting the role of systemic inflammation in disease progression (Chen et al.).

Omelianenko et al. investigated the tumor immune microenvironment (TIME) in thyroid adenoma (TA) and carcinoma (TC) to explore its potential diagnostic value in cytopathology. In a pilot study of 72 cases (36 TA, 36 TC) with histological confirmation and preoperative Bethesda III–V cytology, the authors quantified CD8^+^, CD68^+^, and CD163^+^ immune cells and assessed STAT6 and SMAD4 expression. TC exhibited a highly immunogenic profile with abundant CD8^+^ lymphocytes and macrophages, whereas TA showed low immune infiltration. Immune cell counts in cytology specimens correlated strongly with histological findings. These results suggest that immune cell density in thyroid cytology may serve as an additional criterion for differentiating benign and malignant lesions.

Case report of Yang et al. focuses on a metastatic squamous cell carcinoma of unknown primary (SCCUP) in a 70-year-old female presenting with elevated CA 19–9 and a diaphragmatic mass. Despite extensive evaluation, including PET-CT and a 90-gene expression assay, the primary tumor remained unidentified. The patient underwent surgical resection followed by systemic therapy, achieving 14 months of disease-free survival. This case highlights diagnostic limitations, the potential of multimodal therapy, molecular–clinical discordance, and the need for international collaboration and comprehensive genomic profiling in CUP management.

In therapeutic breakthroughs, a key focus is the multifaceted interplay within the tumor microenvironment (TME) (Anderson and Simon, 2020; Ragunathan et al., 2020). A prominent area involves targeting Tumor-Associated Macrophages (TAMs), which exhibit plasticity, polarizing into M1 (anti-tumor) or M2 (pro-tumor/metastasis promoting) phenotypes (Bai et al.). Therapeutic strategies aim to reprogram M2-TAMs toward the anti-tumor M1 phenotype, utilizing agents like CSF1R inhibitors or blockers targeting the CD47/SIRP*α* axis, or through common drugs like Metformin, which disrupts M2 polarization by activating the AMP-activated protein kinase (AMPK) pathway (Bai et al.). This emphasis on immunomodulation was heavily featured at the 8th Cancer Immunotherapy and Immunomonitoring (CITIM) conference, which highlighted the critical roles of chronic inflammation, Myeloid-Derived Suppressor Cells (MDSCs), and the emerging understanding of the neuro-metabolic-immune regulation of cancer (Rostyslav Bilyy).

Cutting-edge strategies include novel chemical agents designed to induce immunogenic cell death and prolonged immune stimulation (Arkhypov et al., 2025) and delivery systems critical for overcoming treatment obstacles, particularly in challenging diseases like Triple-Negative Breast Cancer (TNBC) (Tiwari et al.). Extracellular vesicles (EVs) are identified both as essential players in promoting TNBC progression and drug resistance (e.g., through carrying EGFR or lncRNA XIST) and as promising drug carriers for targeted therapies due to their low toxicity and ability to traverse biological barriers, Tiwari et al.

Finally, innovation in clinical safety includes the development of a novel sealant combining an absorbable gelatin sponge (mechanical occlusion) with Agkistrodon acutus-derived hemocoagulase (local coagulation, Chen et al.). This dual mechanical and pharmacological barrier significantly reduced the rate of intervention-requiring pneumothorax in pulmonary biopsies (from a literature rate of 22.1% to 2.38%) and introduced a precision-stratified safety protocol based on D-dimer levels to manage hemorrhage risk, Chen et al.

Collectively, these findings underscore a concerted movement toward integrative cancer management, where precise molecular information and sophisticated TME modulation techniques are combined to deliver more effective, personalized, and safer patient care, Alemayehu et al.
